# Classification of HCV NS5B Polymerase Inhibitors Using Support Vector Machine

**DOI:** 10.3390/ijms13044033

**Published:** 2012-03-27

**Authors:** Maolin Wang, Kai Wang, Aixia Yan, Changyuan Yu

**Affiliations:** 1 State Key Laboratory of Chemical Resource Engineering, Department of Pharmaceutical Engineering, Beijing University of Chemical Technology, 15 Bei San Huan East Road, P.O. Box 53, Beijing 100029, China; E-Mails: wangml1240@163.com (M.W.); wangkai0112006@163.com (K.W.); 2 College of Life Science and Technology, Beijing University of Chemical Technology, 15 Bei San Huan East Road, P.O. Box 53, Beijing 100029, China

**Keywords:** hepatitis C virus (HCV), NS5B polymerase inhibitor, classification models, support vector machine (SVM)

## Abstract

Using a support vector machine (SVM), three classification models were built to predict whether a compound is an active or weakly active inhibitor based on a dataset of 386 hepatitis C virus (HCV) NS5B polymerase NNIs (non-nucleoside analogue inhibitors) fitting into the pocket of the NNI III binding site. For each molecule, global descriptors, 2D and 3D property autocorrelation descriptors were calculated from the program ADRIANA.Code. Three models were developed with the combination of different types of descriptors. Model 2 based on 16 global and 2D autocorrelation descriptors gave the highest prediction accuracy of 88.24% and MCC (Matthews correlation coefficient) of 0.789 on test set. Model 1 based on 13 global descriptors showed the highest prediction accuracy of 86.25% and MCC of 0.732 on external test set (including 80 compounds). Some molecular properties such as molecular shape descriptors (InertiaZ, InertiaX and Span), number of rotatable bonds (NRotBond), water solubility (LogS), and hydrogen bonding related descriptors performed important roles in the interactions between the ligand and NS5B polymerase.

## 1. Introduction

Hepatitis C virus (HCV) infection constitutes a global health problem, which affects more than 170 million individuals [1]. According to WHO figures, over 2% of the world population is chronically infected with HCV [2]. Hepatitis C virus (HCV) is a (+)-stranded RNA virus belonging to the Flaviviridae family of enveloped viruses. Previously, the only approved therapy against HCV was pegylated interferon IFNR (IFN), either as monotherapy or in combination with ribavirin. However, this therapy is poorly tolerated and of limited efficacy [3]. The new standard of care (SOC) after recent protease inhibitor approval involves the combination of a protease inhibitor with pegylated-interferon and the oral nucleoside antiviral agent ribavirin [4].

HCV has six major genotype classes, with genotypes 1 and 2 being most prevalent in the United States, Europe, and Japan. Currently combination drug treatment of genotype 2 or 3 is more successful than treatment of genotype 1 infection [5]. HCV is an enveloped single strand RNA virus and encodes a polyprotein chain of about 3000 amino acids, which is processed into structural and non-structural (NS) proteins [6]. Polyprotein processing by viral and cellular host factors results in four structural proteins (Core, E1, E2, p7) and six nonstructural (NS2, NS3, NS4A, NS4B, NS5A, NS5B) proteins.

NS5B is a RNA-dependent RNA polymerase (RdRp) at the core of the HCV replicative complex. As an RNA-dependant RNA polymerase, it has no counterpart in mammalian cells and so it is expected that inhibition of the enzyme will not cause target-related side effects [7]. Given the essential role of this enzyme in viral replication, it is anticipated that agents capable of disrupting its function will prove efficacious in the treatment of HCV infections [8]. Due to its necessary role in viral replication, NS5B RNA-dependent RNA polymerase (RdRp) has been one of the most studied viral protein targets for small molecule HCV therapy [9]. Accordingly, several different structural scaffolds of HCV NS5B inhibitors have been identified and many pharmaceutical companies are competing to identify new drugs. ANA-598, ABT-072 and ABT-333 (the molecular structures of these compounds are not disclosed) have been demonstrated efficacy in phase-II clinical trials [7]. Three specific classes of inhibitors that target the polymerase have been reported. These include nucleoside analogue inhibitors (NIs), non-nucleoside analogue inhibitors (NNIs) and pyrophosphate (PPi) analogues [10].

**Figure 1:**
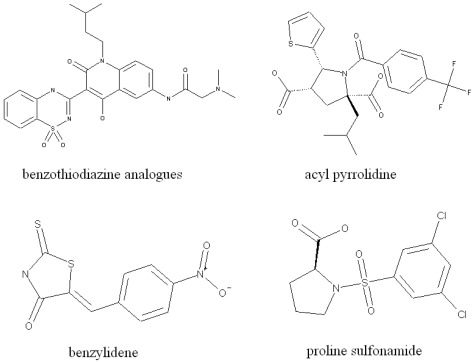
Some representative molecular structures of HCV NS5B NNI III binding site inhibitors.

NNIs (non-nucleoside analogue inhibitors) provide an alternative mechanism to target viral polymerases. Greater variability is possible with HCV inhibitors as multiple allosteric binding sites are present on the HCV polymerase. Up to now, four binding sites of NS5B polymerase have been identified: NNI site I and site II are located in the thumb domain, while III and IV are closer to the active site in the palm domain [10]. In this work, we will work on NS5B NNI III binding site inhibitors. The representative molecules at this binding site are shown in Figure 1.

Recently, Support Vector Machine (SVM) method has been used to build classification models for separating a series of compounds into inhibitors and non-inhibitors [11–13], or high active and low active ligands [14,15] of a protein target to study the structure bioactivity relationship in drug design. The molecular descriptors used for building models can be fast calculated according to the structure of a molecule. So the SVM models could be used for further ligand-based virtual screening from a large compound library [13]. Using ligand-based SVM classification models, only a few potential inhibitors will be chosen for bioassay test. This methodology could reduce the time and cost for new drug discovery.

Lv *et al.* [16] built computational models using several machine learning (ML) methods (support vector machine (SVM), k-nearest neighbor (k-NN), and C4.5 decision tree (C4.5 DT)) for predicting NS5B polymerase inhibitors on a dataset of 1313 compounds, including 552 inhibitors (IC_50_ < 400 nM), 696 non-inhibitors (IC_50_ > 600 nM) and 65 compounds, whose activities range between inhibitors and non-inhibitors (400 nM < IC_50_ < 600 nM). The prediction accuracy for their best model is up to 91.7% for NS5BIs and 78.2% for non-NS5BIs, which was built using a support vector machine (SVM). However, in their models, the HCV NS5B polymerase inhibitors which bind to the different binding sites were put together and were not distinguished.

In this study, a dataset containing 386 NNIs (non-nucleoside analogue inhibitors) fitting into the NNI III binding site of HCV NS5B polymerase, was complied. Each molecule was represented by molecular descriptors calculated from ADRIANA.Code [17]. Using a support vector machine (SVM), three classification models were built to predict whether a compound is active or weakly active as an inhibitor of NS5B polymerase based on a training set containing 266 compounds. And a test set containing 102 compounds was used to validate the models.

## 2. Results and Discussion

### 2.1. Model 1 Built with Global Descriptors

With the descriptor selection method (in Section 3.3), the 27 global descriptors were chosen. From them, 13 descriptors were selected. The 13 selected global descriptors and their correlations with the activity are shown in Table 1.

Then Model 1 was built with the 13 selected global descriptors using SVM. The training set including 266 compounds was used to generate the model, and the test set including 102 compounds was used to test the model. The two parameters of the SVM (*c*, *g*) were selected using the auto-searching program “grid.py” through a fivefold cross-validation in Libsvm. Afterwards, manual selection was done, and the optimum parameters of *c* = 0.00097656, *g* = 8 were selected to build an SVM model. Model 1 had a prediction accuracy of 87.97% on training set, a prediction accuracy of 78.43% and MCC value of 0.625 on test set.

### 2.2. Model 2 with Global Descriptors and 2D Autocorrelation Descriptors

With the descriptor selection method (in Section 3.3), the 27 global descriptors and 88 2D autocorrelation descriptors were chosen. From them, 16 descriptors were selected. The 16 selected global and 2D autocorrelation descriptors and their correlations with the activity are shown in Table 2.

Then Model 2 was built with the 16 selected global and 2D autocorrelation descriptors using SVM. The optimum parameters of *c* = 0.00097656, *g* = 16 were selected to build an SVM model. Model 2 had a prediction accuracy of 95.49% on training set, a prediction accuracy of 88.24% and MCC value of 0.789 on test set.

### 2.3. Model 3 with Global Descriptors and 3D Autocorrelation Descriptors

With the descriptor selection method (in Section 3.3), the 27 global descriptors and 96 3D autocorrelation descriptors were chosen. From them, 19 descriptors were selected. The 19 selected global and 3D autocorrelation descriptors and their correlations with the activity are shown in Table 3.

Then Model 3 was built with the 19 selected global and 3D autocorrelation descriptors using SVM. The optimum parameters of *c* = 0.00097656, *g* = 8 were selected to build an SVM model. Model 3 had a prediction accuracy of 95.11% on training set, a prediction accuracy of 81.37% and MCC value of 0.681 on test set. The results for Model 1,2 and 3 are shown in Table 4.

### 2.4. Relationship between the Selected Molecular Descriptors and Activity

It was found that some molecular properties such as molecular shape descriptors (InertiaZ, InertiaX and Span), number of rotatable bonds (NRotBond) and water solubility (LogS) played significance roles in predicting models, which occurred in each of the three models. Rotatable bonds (NRotBond) can represent the flexibility of a molecule. It can make a difference in the interaction between the ligand and protein. Principal component of the inertia tensor (InertiaX and InertiaZ) of a molecule performed high relevant with the activity.

Moreover, hydrogen bonding donor descriptor HDon (representing number of hydrogen bonding donors derived from the sum of N-H and O-H groups in the molecule.) occurred in both the Model 1 and Model 3, hydrogen bonding acceptor descriptor HAcc (representing number of hydrogen bonding acceptors derived from the sum of nitrogen and oxygen atoms in the molecule.) appeared in Model 1, HAcc_N (Number of hydrogen bonding acceptors derived from the nitrogen atoms in the molecule) and HAcc_O (Number of hydrogen bonding acceptors derived from the oxygen atoms in the molecule) appeared in Model 3. It indicated that the hydrogen-bonding interactions were important for stabilizing the ligand in the HCV NS5B polymerase active centre.

In addition, the 2D autocorrelation for atom charges (σ-, π- and total charge) and atom electronegativities (lone pair electronegativities) appeared in Model 2 (as shown in Table 2); while, the 3D autocorrelation for atom charges (σ-, π- and total charge) and atom electronegativities (lone pair electronegativities) existed in Model 3 (as shown in Table 3). They indicated that the atom charge and electronegativity related descriptors were also important for the interaction between the ligand and NS5B polymerase.

### 2.5. External Test Set

A dataset containing 80 newly synthesized compounds (which also bind to the NNI III binding site of HCV NS5B polymerase) was collected from recent literatures [8,20,21], which were not included in the training and test set. The dataset was used as an external test set, which contains 38 active inhibitors and 42 weakly active inhibitors of NS5B polymerase. The molecular structures can be obtained in the external.sdf in Supporting Information. Prediction performances of the three SVM models are shown in Table 5. Model 1 with 13 selected global descriptors, which gave the best prediction performance on the external test set, had the highest prediction accuracy (Q) of 86.25% and Matthews correlation coefficient (MCC) of 0.732. Both the Model 1 and 2 have SE 92.11%, but their SP are low, which means more weakly inhibitors were wrongly predicted to be active ones by these models. Although Model 2 had higher prediction accuracy on test set, Model 1 showed better prediction accuracy and MCC on external test set than those of Model 2. The reason might be that the molecular structures in the external test set are very different with those in the training set. The training set contains benzothiadiazine analogues, acyl pyrrolidine, benzylidene and proline sulfonamide. The external test set contains benzothiadiazine analogues and acrylic acid derivatives.

One can see that Model 3 showed similar prediction ability with that of Model 1 for the test set. However, compared with Model 1 and Model 2, Model 3 performed poorly on external test set. It was supposed that the molecular structures in external test set are very different with those in the training set. It was observed that acrylic acid derivatives in the external test set were not contained in the training set. It seemed that the 3D descriptors (for building Model 3) were more sensitive than global descriptors (for building Model 1) and 2D descriptors (for building Model 2). As for predicting the activity of the unknown molecules, Model 1 and Model 2 might be of preferred use.

## 3. Experimental Section

### 3.1. Dataset

All compounds used in this work were taken from references [2,22–31]. (All the compounds with their experimental IC_50_ values were contained in our datasets. The molecular structures of the compounds are shown in the Supporting Information). The IC_50_ values of the compounds are those for HCV genotype 1.

The dataset includes 386 HCV NS5B NNI III binding site inhibitors. The activity values of the inhibitors cover a broad range from 2 to 30,000 nM. We considered the compounds with IC_50_ < 400 nM as active and IC_50_ > 600 nM as weakly active [16]. The compounds whose activity (400 nm < IC_50_ < 600 nM) are modest [16], were removed (18 compounds). Accordingly, 217 compounds are active and 151 compounds are weakly active ones. Herein “1” was assigned for active inhibitors and “0” was assigned for weakly active inhibitors. A completed listed of the compounds structures and their corresponding IC_50_ are shown in the file of supporting.sdf in Supporting Information.

We separated the dataset into a training set and a test set. The training/test set selection was done by clustering the compounds based on the fingerprint MACCS [32]. Compounds of similar structural and chemical features were evenly assigned into separate sets using Kohonen’s self-organizing map (calculated with the SONNIA software) [33]. For those compounds without enough structurally and chemically similar counterparts, they were put into the training set. Training set included 266 compounds, which consisted of 169 active compounds and 97 weakly active compounds. Test set included 102 compounds, which consisted of 48 active compounds and 54 weakly active compounds.

Molecular structure building and energy minimization were carried out using the software MOE (Molecular Operation Environment) [32]. The need for computer-generated 3D molecular structures has been recognized in drug design and many other areas. In this work, the optimization of 3D molecular structure was generated by the software CORINA [34].

### 3.2. Molecular Descriptors

A total of 211 descriptors were calculated using ADRIANA.Code [10], including 27 global molecular descriptors (including 8 size and shape descriptors), 88 2D property autocorrelation descriptors and 96 3D property autocorrelation descriptors.

A global molecular descriptor represents each chemical structure by a structural, chemical or physicochemical feature or property of the molecule expressed by a single value. A size and shape descriptor represents a molecule by its 3D structure, and hydrogen atoms are taken into account. For example, the descriptor NRotBond can stand for number of open-chain, single rotatable bonds [35].

The 2D property autocorrelation uses the 2D molecular structure and atom pair properties as a basis to obtain vectorial molecular descriptors. And the atom pair properties are summed up for certain topological distances which count the number of bonds on the shortest path between two atoms. The 2D molecular autocorrelation vectors are calculated by the following equation (Equation (1)):

(1) $$A(d)=\frac{1}{2}\sum_{\begin{array}{l} i,j\\ i\neq j \end{array}}p_{i}p_{j}\delta_{ij}(d-d_{ij})$$

*A(d)* is the topological autocorrelation coefficient referring to atom pairs *i*, *j* which are separated by *d* bonds. *p_i_*, *p_j_* is an atomic property such as partial atomic charge on atom *i* or *j*, respectively. Thus, for each compound, a series of coefficients for different topological distances *d*, a so-called autocorrelation vector is obtained. The 2D molecular autocorrelation vectors were calculated based on the following eight atomic properties: σ charge (SigChg), π charge (PiChg), total charges (TotChg), σ electronegativity (SigEN), π electronegativity (PiEN), lone-pair electronegativity (LpEN), atomic polarizability (Apolariz) and identity (Ident). For each molecule, all the hydrogen atoms were excluded. For each property, the autocorrelation values for eleven distances (0–10 bonds) were calculated. Thus, for each molecule, 88 2D property autocorrelations can be obtained.

Molecules are spatial objects in 3D space. Autocorrelation can also be applied to the 3D structure of a molecule. Thus, the resulting autocorrelation vectors do not only code for the spatial arrangement of the atoms but also for spatial distribution of physicochemical properties in a molecule. Since the distances *d* and *d_ij_* (in Equation (1)) are continuous distances in 3D space between the atoms *i* and *j* (in [Å]), an additional binning of *d* into certain distance intervals (e.g., in steps of 1 Å) is necessary to transform the function *A(d)* into a vector *A(d_n_)* of size *n*.

(2) $$A(d_{n})=\frac{1}{2L_{n}}\sum_{\begin{array}{l} i,j\\ i\neq j \end{array}}p_{i}p_{j}$$

In Equation (2)*L_n_* is the number of distances occurring in a certain distance interval and *p_i_* and *p_j_* are the atom properties of the atoms *i* and *j*. The sampling of all distances in *n* equidistant intervals (e.g., in distance bins of 1–2 Å, 2–3 Å, 3–4 Å, ...) results in an n-dimensional vector of autocorrelation coefficients. The atom property *p* used for the calculation of the 3D autocorrelation coefficients can either be simply the identity (Identity: *p_i_* = *p_j_* = 1) or any physicochemical atom property, such as charge distributions or polarizability effects. (Here, eight atom properties as above were computed). For each molecule, all the hydrogen atoms were excluded. For each of the eight properties, a series of 12 vectors were computed, where *L_n_* correspond to the 12 3D distance intervals from 1–2 Å, 2–3 Å, … to 12–13 Å. Thus for each molecule, 96 3D properties autocorrelations can be obtained.

### 3.3. Descriptors Selection

Pearson correlation analysis [36] can reduce descriptors that are not significantly correlated with activity and highly correlated with each other. In this work, descriptors whose Pearson correlation coefficient with activity was less than 0.15 were deleted. If the pairwise correlation coefficient between any two descriptors was higher than 0.85, the descriptor that had a lower correlation coefficient with the activity was removed. Then a stepwise variable selection method was carried out. All the descriptors selected by correlation analysis were sorted in a descending order according to their correlation coefficient with activity. Simple linear regression of activity and the first descriptor was done to obtain an initial equation. Other descriptors were then added to the regression equation one by one. A significance test was carried out for each new regression equation and every descriptor in the equation. If the new regression equation was not “statistically significant” because of the addition of a new descriptor, that the new descriptor was removed. Descriptors that are not “statistically significant” in the equation were also removed, followed by construction of a new regression equation. The process was continued until no descriptor can be added or removed by this approach. Before training, the input data (selected descriptors) were scaled to a [0.1, 0.9] range via the Equation (3).

(3) $$x_{i}^{*}=\frac{x_{i}-x_{\text{min}}}{x_{\text{max}}-x_{\text{min}}}\times 0.8+0.1$$

where *X_i_* was the original value, and *X*_i_^*^ was the scaled value. *X*_min_ and *X*_max_ were the corresponding minimum and maximum values of the descriptor variable, respectively.

### 3.4. Support Vector Machine

The SVM [37] technique was applied to build the classification models of NS5B polymerase inhibitors. SVM is a useful tool for classification. It is based on the Vapnik Chervonenkis dimension and Vapnik’s Structural Risk Minimization principle. Its main idea is to map data into a high-dimensional space in which a constrained quadratic programming problem will be solved and a separating hyperplane with the maximal margin will be found.

In this study, the Libsvm [38] program was utilized to build SVM models. Libsvm software is developed by Chang and Lin for SVM analysis. This software is based on the function of classification. There are four basic kernels in Libsvm software. The commonly used kernel, the Radial Basis Function (RBF) kernel (Equation (4)), was used. It is used to convert the data into a higher-dimensional space. The parameters *c* (Equation (5)) and g were chosen by the auto-searching program “grid.py” through a cross-validation method.

(4) k(x,y)=exp(-g||x-y||2)

(5) $$\begin{array}{l} \underset{w,b,\xi }{\text{min}}\frac{1}{2}W^{T}W+C\sum_{i=1}^{1}\xi_{i}\\ Subject\;to\;\;\;y_{i}(W^{T}\varphi (X_{i})+b)\geq 1-\xi_{i}\\ \xi_{i}\geq 0 \end{array}$$

### 3.5. Evaluation of Models

Several methods were employed to evaluate the performance of the classification models, such as sensitivity (SE), specificity (SP), the overall prediction accuracy (Q) and Matthews correlation coefficient (MCC), which are listed by Equations (6–9). True positives (TP) stands for the number of active inhibitors which were predicted as active inhibitors, true negatives (TN) stands for the number of weakly active inhibitors which were predicted as weakly active inhibitors, false positives (FP) stands for the number of weakly active inhibitors which were predicted as active inhibitors and false negatives (FN) stands for the number of active inhibitors which were predicted as weakly active inhibitors. SE stands for the prediction accuracy for active inhibitors and SP stands for the prediction accuracy for weakly active inhibitors, respectively.

(6) SE=(TP/(TP+FN))×100%

(7) SP=(TN/(TN+FP))×100%

(8) Q=((TP+TN)/(TP+TN+FP+FN))×100%

(9) $$MCC=\frac{TP\times TN-FN\times FP}{\sqrt{\left(TP+FN)(TP+FP)(TN+FN)(TN+FP\right)}}$$

According to Equation (9), a higher MCC value means a better prediction performance.

## 4. Conclusions

In this study, three SVM models for classifying hepatitis C virus (HCV) NS5B NNI III binding site inhibitors were developed. All the three models showed good prediction ability for the test set. As for predicting the activity of the unknown molecular structures, Model 1 and Model 2 might be of preferred use.

It was found that some molecular properties such as molecular shape descriptors (InertiaZ, InertiaX and Span), number of rotatable bonds (NRotBond), water solubility (LogS), and hydrogen bonding related descriptors performed important roles in the interactions between the ligand and NS5B polymerase. In addition, the atom charges and atom electronegativities related descriptors were also important.

## Figures and Tables

**Table 1:** The intercorrelations between the 13 selected global descriptors and the activity^a^.

	Activity	InertiaZ	HAcc	NAtoms	NViolationsRo5	LogS	InertiaX	Span	HDon	HDon_N	NRotBond	RComplexity	Dipole
InertiaZ	0.540	1											
HAcc	0.492	0.794	1										
NAtoms	0.457	0.812	0.841	1									
NViolationsRo5	0.452	0.770	0.734	0.607	1								
LogS	−0.439	−0.735	−0.404	−0.607	−0.475	1							
InertiaX	0.426	0.830	0.720	0.822	0.673	−0.721	1						
Span	0.403	0.824	0.677	0.760	0.501	−0.625	0.667	1					
HDon	0.397	0.629	0.724	0.655	0.515	−0.241	0.569	0.523	1				
HDon_N	0.364	0.653	0.604	0.503	0.553	−0.314	0.460	0.576	0.792	1			
NRotBond	0.323	0.686	0.681	0.759	0.615	−0.431	0.702	0.658	0.461	0.348	1		
RComplexity	0.298	0.280	0.426	0.497	−0.007	−0.246	0.266	0.483	0.366	0.259	0.077	1	
Dipole	0.296	0.303	0.416	0.464	0.067	−0.306	0.322	0.534	0.288	0.237	0.179	0.794	1
Eccentric	0.168	0.275	0.139	−0.039	0.189	−0.018	−0.241	0.267	0.089	0.374	−0.045	0.009	−0.008

a InertiaZ represents principal component of the inertia tensor in z-direction; HAcc represents number of hydrogen bonding acceptors derived from the sum of nitrogen and oxygen atoms in the molecule; NAtoms represents number of all atoms in the molecule; NViolationsRo5 represents number of violations of the Lipinski's rule of 5 (Weight > 500, XlogP > 5, HDon > 5, HAcc > 10); LogS represents solubility of the molecule in water in [log units]; InertiaX represents principal component of the inertia tensor in *x*-direction; Span represents radius of the smallest sphere centered at the center of mass which completely encloses all atoms in the molecule; HDon represents number of hydrogen bonding donors derived from the sum of N-H and O-H groups in the molecule; HDon_N represents number of hydrogen bonding donors N-H groups in the molecule; NRotBond represents number of open-chain, single rotatable bonds; RComplexity represents ring complexity according to the approach by J. Gasteiger and C. Jochum [18]; Dipole represents dipole moment in [Debye] of the molecule; Eccentric represents molecular eccentricity [19].

**Table 2:** The correlation coefficients between the 16 selected global and 2D autocorrelation descriptors and the activity.

	Activity	Description of Selected Descriptors
Span	0.403	Radius of the smallest sphere centered at the center of mass which completely encloses all atoms in the molecule
NRotBond	0.323	Number of open-chain, single rotatable bonds
LogS	−0.439	Solubility of the molecule in water in [log units]
InertiaZ	0.540	Principal component of the inertia tensor in *z*-direction
InertiaX	0.426	Principal component of the inertia tensor in *x*-direction
2DACorr_TotChg_11	−0.277	The eleventh component of 2D autocorrelation coefficients for σ and π charges, where the distance *d* = 10
2DACorr_TotChg_1	0.523	The first component of 2D autocorrelation coefficients for σ and π charges, where the distance *d* = 0
2DACorr_SigChg_4	−0.452	The fourth component of 2D autocorrelation coefficients for σ charge, where the distance *d* = 3
2DACorr_SigChg_3	0.272	The third component of 2D autocorrelation coefficients for σ charge, where the distance *d* = 2
2DACorr_SigChg_2	−0.249	The second component of 2D autocorrelation coefficients for σ charge, where the distance *d* = 1
2DACorr_PiChg_10	0.326	The tenth component of 2D autocorrelation coefficients for π charges, where the distance *d* = 9
2DACorr_LpEN_8	0.305	The eighth component of 2D autocorrelation coefficient for lone pair electronegativities, where the distance *d* = 7
2DACorr_LpEN_6	0.582	The sixth component of 2D autocorrelation coefficient for lone pair electronegativities, where the distance *d* = 5
2DACorr_LpEN_4	0.198	The fourth component of 2D autocorrelation coefficient for lone pair electronegativities, where the distance *d* = 3
2DACorr_LpEN_10	0.166	The tenth component of 2D autocorrelation coefficient for lone pair electronegativities, where the distance *d* = 9
2DACorr_Ident_11	0.421	The eleventh component of 2D autocorrelation coefficient for identity, where the distance *d* = 10

**Table 3:** The correlation coefficients between the selected 19 global and 3D autocorrelation descriptors and the activity.

	Activity	Description of Selected Descriptors
HDon	0.397	Number of hydrogen bonding donors derived from the sum of N-H and O-H groups in the molecule
HAcc_N	0.431	Number of hydrogen bonding acceptors derived from the nitrogen atoms in the molecule
HAcc_O	0.417	Number of hydrogen bonding acceptors derived from the oxygen atoms in the molecule
LogS	−0.439	Solubility of the molecule in water in [log units]
NRotBond	0.323	Number of open-chain, single rotatable bonds
InertiaX	0.426	Principal component of the inertia tensor in *x*-direction
InertiaZ	0.540	Principal component of the inertia tensor in *z*-direction
Span	0.403	Radius of the smallest sphere centered at the center of mass which completely encloses all atoms in the molecule
Eccentric	0.168	Molecular eccentricity [19]
3DACorr_SigChg_2	−0.210	3D autocorrelation weighted by σ atom charges, where d is in the range of 2–3 Å
3DACorr_SigChg_6	−0.364	3D autocorrelation weighted by σ atom charges, where d is in the range of 6–7 Å
3DACorr_SigChg_7	0.345	3D autocorrelation weighted by σ atom charges, where d is in the range of 7–8 Å
3DACorr_PiChg_4	−0.165	3D autocorrelation weighted by π atom charges, where d is in the range of 4–5 Å
3DACorr_PiChg_10	0.166	3D autocorrelation weighted by π atom charges, where d is in the range of 10–11 Å
3DACorr_TotChg_1	−0.514	3D autocorrelation weighted by total atom charges (sum of σ, π charges), where d is in the range of 1–2 Å
3DACorr_TotChg_7	0.348	3D autocorrelation weighted by total atom charges (sum of σ, π charges), where d is in the range of 7–8 Å
3DACorr_PiEN_7	0.436	3D autocorrelation weighted by π atom electronegativities, where d is in the range of 7–8 Å
3DACorr_LpEN_5	0.413	3D autocorrelation weighted by lone pair electronegativities, where d is in the range of 5–6 Å
3DACorr_LpEN_12	0.350	3D autocorrelation weighted by lone pair electronegativities, where d is in the range of 12–13 Å

**Table 4:** Prediction performance of the three SVM models^a^.

Model	Number of Descriptors	Number of Compounds	Training Set	Test Set			

		Training Set/Test Set	Accuracy	SE b	SP c	Accuracy	MCC d
Model 1	13	266/102	87.97%	97.92%	61.11%	78.43%	0.625
Model 2	16	266/102	95.49%	100%	77.78%	88.24%	0.789
Model 3	19	266/102	95.11%	100%	64.81%	81.37%	0.681

a Model 1 represents the model built with 13 selected global descriptors as shown in Table 1. Model 2 represents the model built with 16 selected global and 2D autocorrelation descriptors as shown in Table 2. Model 3 represents the model built with 19 selected global and 3D autocorrelation descriptors as shown in Table 3;

b SE (sensitivity) represents the prediction accuracy of the active inhibitors;

c SP (specificity) represents the prediction accuracy of the weakly active inhibitors;

d MCC represents Matthews Correlation Coefficient.

**Table 5:** Prediction accuracy on the external test set with three models a. The external test set contains 38 active inhibitors and 42 weakly active inhibitors of NS5B polymerase.

Model	Number of Descriptors	Number of Compounds	SE b	SP c	Accuracy	MCC d
Model 1	13	80	92.11%	80.95%	86.25%	0.732
Model 2	16	80	92.11%	69.05%	80.00%	0.623
Model 3	19	80	65.79%	54.76%	60.00%	0.206

a Model 1 represents the model built with 13 selected global descriptors as shown in Table 1. Model 2 represents the model built with 16 selected global and 2D Autocorrelation descriptors as shown in Table 2. Model 3 represents the model built with 19 selected global and 3D Autocorrelation descriptors as shown in Table 3;

b SE (sensitivity) represents the prediction accuracy of the active inhibitors;

c SP (specificity) represents the prediction accuracy of the weakly active inhibitors;

d MCC represents Matthews Correlation Coefficient.
